# Serum N-Terminal Pro-B-Type Natriuretic Peptide Level is Negatively Associated with Vascular Reactivity Index by Digital Thermal Monitoring in Patients with Hypertension

**DOI:** 10.31083/j.rcm2506214

**Published:** 2024-06-14

**Authors:** Chien-Hao Hsiao, Chiu-Fen Yang, Ji-Hung Wang, Bang-Gee Hsu

**Affiliations:** ^1^Division of Cardiology, Hualien Tzu Chi Hospital, Buddhist Tzu Chi Medical Foundation, 97004 Hualien, Taiwan; ^2^School of Medicine, Tzu Chi University, 97004 Hualien, Taiwan; ^3^Division of Nephrology, Hualien Tzu Chi Hospital, Buddhist Tzu Chi Medical Foundation, 97004 Hualien, Taiwan

**Keywords:** N-terminal pro-B-type natriuretic peptide, hypertension, vascular reactivity index, endothelial dysfunction

## Abstract

**Background:**

B-type natriuretic peptide (BNP) coordinates endothelial homeostasis and remodeling, with endothelial dysfunction associated with cardiovascular mortality in the general population without heart failure. The objective of this study was to investigate the correlation between serum N-terminal pro-B-type natriuretic peptide (NT-pro-BNP) levels and endothelial dysfunction among patients diagnosed with hypertension.

**Methods:**

This cross-sectional, single-center study included 90 patients with hypertension. An electrochemiluminescence immunoassay measured NT-pro-BNP levels, and a digital thermal monitoring device calculated a vascular reactivity index (VRI) as a measurement for endothelial function. In this study, VRI < 1.0 denoted poor vascular reactivity, 1.0 ≤ VRI < 2.0 indicated intermediate vascular reactivity, and a VRI ≥ 2.0 suggested good vascular reactivity.

**Results:**

Out of all the hypertensive patients, eight (8.9%) displayed poor vascular reactivity (VRI < 1.0), while 39 (43.3%) exhibited intermediate vascular reactivity (1.0 ≤ VRI < 2.0), leaving the remaining 43 patients demonstrating good vascular reactivity. Older age (*p* = 0.012) and elevated serum NT-pro-BNP levels (*p* < 0.001) were found to be associated with poorer vascular reactivity. Older age (*r* = –0.221, *p* = 0.036) and log-transformed serum levels of NT-pro-BNP (log-NT-pro-BNP, *r* = –0.505, *p* < 0.001) exhibited a negative correlation with VRI values in patients with hypertension. Following a multivariate linear regression test, serum log-NT-pro-BNP level (β = –0.505, adjusted $R^{2}$ change = 0.246, *p* < 0.001) emerged as being significantly and independently associated with VRI values among hypertensive patients.

**Conclusions:**

In patients with hypertension, there was a negative association observed between serum log-NT-pro-BNP levels and endothelial dysfunction determined by VRI values.

## 1. Introduction

Hypertension stands out as the primary risk factor for cardiovascular disease worldwide, with an estimated one-third of adults aged 30–79 years affected, as reported in 2019 [1]. Uncontrolled hypertension significantly contributes to coronary artery disease (CAD), stroke, heart failure, chronic kidney disease, peripheral artery disease, and atrial fibrillation [2, 3, 4, 5, 6, 7], leading to premature morbidity and mortality in affected individuals. Endothelial dysfunction, characterized by changes in vascular endothelial properties such as vasoconstriction, hyperpermeability, and pro-inflammatory/prothrombotic status, may elevate peripheral vascular resistance, enhance vascular remodeling, and elevate blood pressure [8]. Conversely, the activated renin-angiotensin-aldosterone system (RAAS) and sympathetic nervous system (SNS) in patients with hypertension also contribute to endothelial dysfunction by altering shear stress and increasing oxidative stress, creating a pathogenic cycle and subsequent atherosclerotic cardiovascular disease [8, 9, 10].

N-terminal pro-B-type natriuretic peptide (NT-pro-BNP), a biologically inert byproduct of pro-B-type natriuretic peptide (pro-BNP) cleavage at its N-terminal end, exhibits a longer degradation time than B-type natriuretic peptide (BNP). Apart from its established roles in diagnosing, risk-stratifying, and prognosticating acute or chronic heart failure [11, 12], plasma BNP and NT-pro-BNP levels also predict the risk of mortality and cardiovascular events in asymptomatic populations without heart failure [13, 14, 15]. Even after adjusting for echocardiographic indices of possible preclinical cardiac remodeling, such as left ventricular mass, diastolic dysfunction, and left ventricular ejection fraction, the association between natriuretic peptides and cardiovascular mortality remains significant [16]. Considering that endothelial dysfunction plays a critical role in the onset and progression of atherosclerotic disease and is linked to an elevated risk of cardiovascular disease [8, 17], there is considerable interest in elucidating the association between NT-pro-BNP and endothelial dysfunction in patients with hypertension. In this cross-sectional study, we aimed to determine the correlation between serum NT-pro-BNP levels and endothelial dysfunction in participants with hypertension.

## 2. Materials and Methods

### 2.1 Participants

Ninety Taiwanese patients with hypertension who visited the cardiovascular outpatient department of Hualien Tzu Chi Medical Center were recruited from October 2016 to May 2017. Medical records of all patients were reviewed, and CAD was defined as having more than 50% stenosis in any significant epicardial coronary artery. Hypertension was diagnosed according to the Eighth Joint National Committee (JNC 8) guidelines, characterized by systolic blood pressure (SBP) ≥140 mmHg and diastolic blood pressure (DBP) ≥90 mmHg or the use of any antihypertensive agents within the preceding fourteen days [18]. Participants were considered to have diabetes mellitus (DM) if their fasting blood sugar levels were ≥126 mg/dL or if they were taking oral hypoglycemic agents or insulin injections. Informed consent was obtained from all participants before the investigation. Exclusion criteria included an inability to provide informed consent, acute infections, acute coronary syndrome, active cancer, limb amputation, uncontrolled arrhythmia, or heart failure during blood sampling. The Research Ethics Committee of Hualien Tzu Chi Hospital, Buddhist Tzu Chi Medical Foundation, approved this study.

### 2.2 Anthropometric Measurements and Biochemical Investigations

Participants were instructed to fast for 8–12 hours before the investigation. Anthropometric variables, including weight and height, were recorded, and body mass index was calculated by dividing weight (kg) by the square of height ($m^{2}$). After resting for at least 10 minutes, the morning blood pressure was assessed twice by trained staff, with measurements taken at 5-minute intervals, and the average was calculated for subsequent analysis. Fasting serum levels of total cholesterol, triglycerides, high-density lipoprotein cholesterol (HDL-C), low-density lipoprotein cholesterol (LDL-C), fasting glucose, albumin, blood urea nitrogen (BUN), and creatinine were measured using an autoanalyzer (Siemens Advia 1800; Siemens Healthcare, Henkestr, Germany). Serum NT-pro-BNP levels were assayed by electrochemiluminescence immunoassay on an Elecsys 2010 Immunoanalyzer (Roche Diagnostics, Indianapolis, IN, USA) [19, 20]. The chronic Kidney Disease (CKD) Epidemiology Collaboration creatine equation was used to calculate the estimated glomerular filtration rate (eGFR).

### 2.3 Endothelial Function Measurements

Participants had to abstain from using cigarettes, alcohol or caffeinated drinks, and vasoactive agents before endothelial function measurement, which was determined using an Food and Drug Administration (FDA)-approved digital thermal monitoring (DTM) device (Endothelix, Houston, TX, USA). Before measurement, patients were required to recline for 15 min in a temperature-controlled laboratory room maintained at approximately 24 °C. A blood pressure cuff was placed on the patient’s right upper arm, while sensors detecting temperature change were placed on the index fingers, with the left side designated as the control. The DTM of both hands was measured following a stable period of 5 min, then the cuff was rapidly inflated to a level 50 mmHg higher than the SBP, which wassustained for 5 min. It wasthen deflated to activate hyperemia in the fingertips. A more significant rebound in temperature indicated enhanced reactivity in vascular response. The vascular reactivity index (VRI) was determined by the maximum temperature difference observed between the rebound and baseline curves during the reactive hyperemia phase. Values were determined by VENDYS-II software (Endothelix Inc., Houston, TX, USA). The typical range for VRI values ranged from 0.0 to 3.5. These values were grouped as poor (values <1.0), intermediate (values between 1.0 and 1.9), and good (values >2.0) [21, 22, 23, 24]. Poor and intermediate VRI values were collectively regarded as vascular reactivity dysfunction.

### 2.4 Statistical Analyses

The Kolmogorov–Smirnov test was used to test the normal distribution of the data. Continuous variables with nonparametric distribution were analyzed using the Kruskal–Wallis test, while the one-way analysis of variance test was used for continuous variables with normal distribution. Parameters such as fasting blood sugar, triglycerides, BUN, creatinine, and NT-pro-BNP levels underwent a logarithmic transformation to achieve normal distribution. Correlation between clinical and laboratory variables and VRI values was analyzed using simple linear and multivariate regression models. Univariate and multivariate logistic regression analyses examined the association between NT-pro-BNP levels and poor vascular reactivity or vascular reactivity dysfunction. Statistical significance was defined as a *p*-value less than 0.05. All statistical analyses were conducted using SPSS software, version 19.0 (IBM Corp., Armonk, NY, USA).

## 3. Results

The clinical characteristics and biochemistry data of the 90 hypertensive patients are presented in Table 1. In the study cohort, 37 patients (41.1%) had DM, 68 (75.6%) had CAD, and 17 (17.8%) were smokers. Regarding VRI values, 43 (47.8%), 39 (43.3%), and 8 (8.9%) subjects had good, intermediate, and poor VRI values, respectively. Older age (*p* = 0.012) and higher NT-pro-BNP levels (*p* < 0.001) significantly differed in the groups with worse VRI values. There were no significant between-group differences in the remaining variables, including sex, smoking status, history of DM or CAD, and use of antihypertensive or anti-lipid agents.

**Table 1.** **Clinical characteristics of study subjects stratified by different grading of vascular reactivity index**.

Characteristics	Total population (n = 90)	Good vascular reactivity (n = 43)	Intermediate vascular reactivity (n = 39)	Poor vascular reactivity (n = 8)	*p*-value
Age (years)	61.48 ± 7.66	60.08 ± 5.76	61.54 ± 8.58	68.71 ± 8.69	0.012*
Body weight (kg)	73.58 ± 12.03	71.62 ± 9.32	76.65 ± 14.27	69.09 ± 10.76	0.090
Height (cm)	164.19 ± 7.34	162.84 ± 7.86	165.47 ± 7.22	165.25 ± 3.20	0.247
Body mass index (kg/$m^{2}$)	27.23 ± 3.70	27.03 ± 3.23	27.88 ± 4.17	25.21 ± 3.25	0.158
VRI	1.92 ± 0.60	2.40 ± 0.35	1.65 ± 0.22	0.70 ± 0.25	<0.001*
SBP (mmHg)	136.97 ± 17.90	138.49 ± 16.72	137.05 ± 19.30	128.38 ± 16.54	0.344
DBP (mmHg)	81.09 ± 10.26	82.28 ± 9.90	81.00 ± 10.68	75.13 ± 9.09	0.195
Total cholesterol (mg/dL)	163.37 ± 40.69	161.95 ± 33.60	160.41 ± 42.52	185.38 ± 62.00	0.276
Triglyceride (mg/dL)	133.50 (100.50–207.50)	131.00 (101.00–209.00)	135.00 (110.00–220.00)	142.50 (81.25–187.25)	0.854
HDL-C (mg/dL)	45.84 ± 10.25	47.14 ± 10.85	43.92 ± 9.96	48.25 ± 7.34	0.290
Fasting glucose (mg/dL)	113.00 (92.00–153.25)	114.00 (92.00–155.00)	114.00 (94.00–153.00)	98.50 (86.25–148.25)	0.494
LDL-C (mg/dL)	91.26 ± 32.08	90.14 ± 29.90	88.41 ± 28.51	111.13 ± 52.94	0.181
BUN (mg/dL)	17.00 (14.00–19.00)	16.00 (14.00–19.00)	17.00 (14.00–20.00)	18.00 (13.25–23.50)	0.465
Creatinine (mg/dL)	1.00 (0.88–1.10)	1.00 (0.88–1.10)	1.00 (0.90–1.10)	1.00 (0.90–1.30)	0.164
Albumin (mg/dL)	4.37 ± 0.24	4.41 ± 0.25	4.35 ± 0.19	4.26 ± 0.33	0.207
eGFR (mL/min)	81.65 ± 20.93	86.15 ± 21.96	77.88 ± 18.30	75.85 ± 22.82	0.145
NT-pro-BNP (pg/mL)	153.11 (67.53–249.56)	95.53 (43.26–188.59)	176.63 (73.36–250.17)	533.74 (403.89–794.86)	<0.001*
Male, n (%)	77 (85.6)	35 (81.4)	35 (89.7)	7 (87.5)	0.554
Diabetes mellitus, n (%)	37 (41.1)	14 (32.6)	18 (46.2)	5 (62.5)	0.200
CAD, n (%)	68 (75.6)	32 (74.4)	30 (76.9)	6 (75.0)	0.965
Smoking, n (%)	16 (17.8)	10 (23.3)	5 (12.8)	1 (12.5)	0.429
ACE inhibitor user, n (%)	17 (18.9)	7 (16.3)	9 (23.1)	1 (12.5)	0.654
ARB user, n (%)	48 (53.3)	25 (58.1)	18 (46.2)	5 (62.5)	0.478
β-blocker user, n (%)	40 (44.4)	17 (39.5)	18 (46.2)	5 (62.5)	0.467
CCB user, n (%)	46 (51.1)	23 (53.5)	20 (51.3)	3 (37.5)	0.708
Statin user, n (%)	69 (76.7)	31 (72.1)	32 (82.1)	6 (75.0)	0.563
Fibrate user, n (%)	6 (6.7)	3 (7.0)	2 (5.1)	1 (12.5)	0.744

Continuous variables are expressed as means and SD and tested by one-way ANOVA; non-parametric variables are expressed as medians and IQR then analysed by Kruskal-Wallis test; values presented as number (%) are analyzed by the chi-square test. VRI, vascular reactivity index; SBP, systolic blood pressure; DBP, diastolic blood pressure; BUN, blood urea nitrogen; eGFR, estimated glomerular filtration rate; HDL-C, high-density lipoprotein cholesterol; LDL-C, low-density lipoprotein cholesterol; NT-pro-BNP, N-terminal pro-B-type natriuretic peptide; CAD, coronary artery disease; ACE, angiotensin-converting enzyme; ARB, angiotensin-receptor blocker; CCB, calcium-channel blocker; SD, standard deviation; IQR, interquartile range; ANOVA, analysis of variance. *p < 0.05 was defined as statistically significant.

The analysis by multivariate logistic regression (Model 3) after adjusting for other confounder factors found that NT-pro-BNT was significantly and independently associated with vascular reactivity dysfunction, with an odds ratio (OR) of 1.008 (95% confidence interval [CI] = 1.003–1.012; *p* = 0.001). It was also significantly and independently correlated with poor vascular reactivity, with an OR of 1.015 (95% CI = 1.003–1.026, *p *= 0.014) in patients with hypertension (Table 2).

**Table 2.** **Vascular reactivity dysfunction or poor vascular reactivity tested by multivariate logistic regression model**.

Model	NT-pro-BNP (per 1 pg/mL of elevation) for vascular reactivity dysfunction		NT-pro-BNP (per 1 pg/mL of elevation) for poor vascular reactivity	
	OR (95% CI)	*p*-value	OR (95% CI)	*p*-value
Unadjusted model	1.007 (1.003–1.011)	0.001*	1.008 (1.004–1.013)	<0.001*
Model 1	1.007 (1.003–1.012)	0.001*	1.010 (1.004–1.016)	0.001*
Model 2	1.008 (1.003–1.012)	0.001*	1.013 (1.004–1.022)	0.006*
Model 3	1.008 (1.003–1.012)	0.001*	1.015 (1.003–1.026)	0.014*

Vascular reactivity dysfunction includes intermediate and poor vascular reactivity. Model 1: adjusted for age, sex, and body mass index. Model 2: Adjusted for Model 1 plus systolic blood pressure, diastolic blood pressure, and eGFR. Model 3: Adjusted for Model 2 plus albumin, fasting glucose, total cholesterol, triglyceride, HDL-C, and LDL-C. HDL-C, high-density lipoprotein cholesterol; LDL-C, low-density lipoprotein cholesterol; eGFR, estimated glomerular filtration rate; NT-pro-BNP, N-terminal pro-B-type natriuretic peptide; OR, odds ratio; CI, confidence interval. *p < 0.05 was considered statistically significant.

**Fig. 1.**
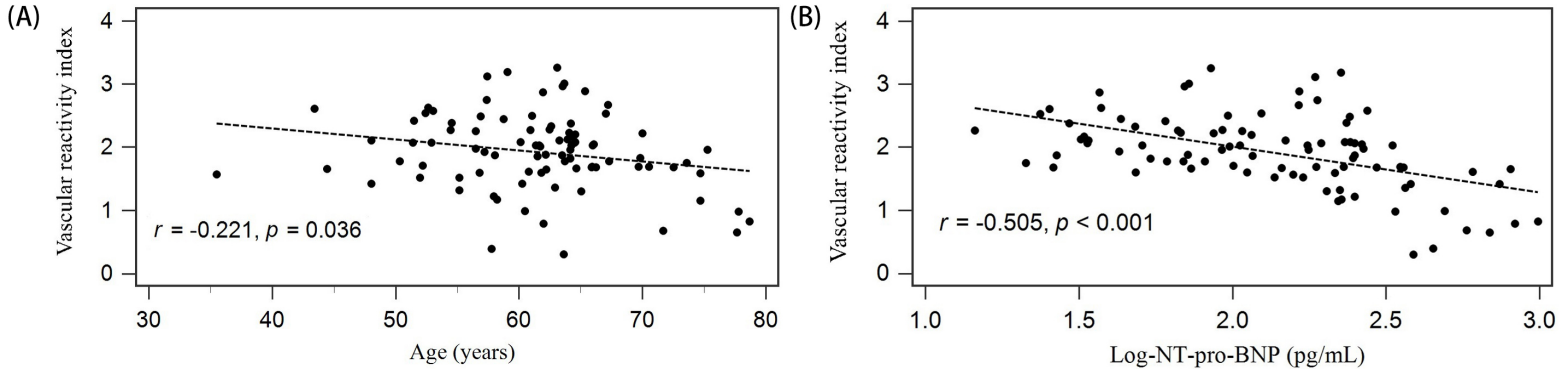
**Association between vascular reactivity index and (A) age, (B) serum logarithmically transformed N-terminal pro-B-type natriuretic peptide (log-NT-pro-BNT) levels among 90 hypertensive patients**.

The results of the analysis by simple or multivariate linear regression are shown in Table 3. The significant variables associated with VRI values were older age (*r* = –0.221, *p* = 0.036) and serum logarithmically transformed NT-pro-BNT levels (log-NT-pro-BNT, *r* = –0.505, *p* < 0.001) with a negative relationship. After adjusting for these significant variables from linear regression analysis with a multivariable stepwise approach, a low serum level of log-NT-pro-BNT (β = –0.505, adjusted *$R^{2}$* change = 0.246, *p* < 0.001) was significantly and independently associated with VRIs in patients with hypertension. The scattered graphs of VRI values with age and serum log-NT-pro-BNT levels in the study cohort are illustrated in Fig. 1A,B.

**Table 3.** **Correlation of clinical and laboratory variables with vascular reactivity index levels analyzed by regression model among 90 hypertensive patients**.

Variables	Vascular reactivity index				
	Simple linear regression model		Multivariable regression model		
	*r*	*p*-value	Adjusted $R^{2}$ change	Beta	*p*-value
Male	0.090	0.401	**—**	**—**	**—**
Coronary artery disease	0.097	0.364			
Diabetes mellitus	–0.087	0.417	**—**	**—**	**—**
Smoking	0.116	0.278	**—**	**—**	**—**
Age (years)	–0.221	0.036*	**—**	**—**	**—**
DBP (mmHg)	0.185	0.081	**—**	**—**	**—**
SBP (mmHg)	0.147	0.167	**—**	**—**	**—**
Height (cm)	–0.134	0.208	**—**	**—**	**—**
Body mass index (kg/$m^{2}$)	0.002	0.982	**—**	**—**	**—**
Body weight (kg)	–0.080	0.455	**—**	**—**	**—**
Total cholesterol (mg/dL)	–0.187	0.077	**—**	**—**	**—**
LDL-C (mg/dL)	–0.151	0.156	**—**	**—**	**—**
Log-Triglyceride (mg/dL)	–0.051	0.634	**—**	**—**	**—**
HDL-C (mg/dL)	0.068	0.527			
Log-Glucose (mg/dL)	0.106	0.319	**—**	**—**	**—**
Albumin (mg/dL)	0.194	0.066	**—**	**—**	**—**
eGFR (mL/min)	0.119	0.263	**—**	**—**	**—**
Log-BUN (mg/dL)	–0.144	0.175	**—**	**—**	**—**
Log-Creatinine (mg/dL)	–0.141	0.185	**—**	**—**	**—**
Log-NT-pro-BNP (pg/mL)	–0.505	<0.001*	0.246	–0.505	<0.001*

Log transformation before analysis was performed for serum triglyceride levels, fasting glucose, blood urea nitrogen, creatinine, and NT-pro-BNP due to non-normal distribution. Simple linear regression model or multivariable linear regression model (adjusted by age and log-NT-pro-BNP) was conducted to analyze data. SBP, systolic blood pressure; DBP, diastolic blood pressure; BUN, blood urea nitrogen; eGFR, estimated glomerular filtration rate; NT-pro-BNP, N-terminal pro-B-type natriuretic peptide; LDL-C, low-density lipoprotein cholesterol; HDL-C, high-density lipoprotein cholesterol. *p < 0.05 was defined as statistically significant.

## 4. Discussion

In this study of patients with hypertension, our analysis revealed that older age and higher serum NT-pro-BNP levels correlated with a poor VRI determined by the DTM test. After adjusting for significant variables, serum log-NT-pro-BNP levels exhibited an independent and significant association with VRI values among patients with hypertension.

The vascular endothelium constitutes the innermost layers of arteries, capillaries, and veins. It serves as a semipermeable barrier between the bloodstream and vascular smooth muscle layers and plays a pivotal role in regulating vascular tone and homeostasis [25]. Endothelial dysfunction, characterized by an imbalance in regulation, encompasses reduced endothelium-dependent dilation (EDD), inflammation, and increased thrombosis propensity [26]. Advancing age has been strongly linked to reduced EDD, as evidenced by flow-mediated dilation of the brachial artery in the Framingham population [27]. This phenomenon observed in aging vascular endothelium can be attributed to various factors, including diminished nitric oxide bioavailability, elevated endothelin-1 production, inadequate tetrahydrobiopterin availability, and heightened oxidative stress due to increased reactive oxygen species generation [26, 28]. In our previous study, older age emerged as an independent predictor of lower VRI in patients with CAD [29]. Consistently, our present study also demonstrates a significant association between older age and poorer VRI in hypertensive patients.

In physiological and pathological conditions, BNP is released from cardiac ventricles in response to increased intravascular volume or intramural pressure. Physiologically, BNP enhances renal perfusion and natriuresis, inhibiting the SNS and RAAS and exerting antifibrotic and antihypertrophic effects on cardiomyocytes [30]. Additionally, BNP demonstrates vasodilatory effects by elevating cyclic guanosine monophosphate and anti-inflammatory effects by increasing plasma adiponectin levels, suggesting its role in regulating endothelial function [30, 31]. Clinical studies have highlighted the predictive role of natriuretic peptides in endothelial dysfunction, preclinical vascular remodeling, and clinical atherosclerotic disease. In a cross-sectional study involving participants without preexisting atherosclerotic disease, plasma BNP levels were independently correlated with endothelial dysfunction determined by acetylcholine-induced vasodilation [32]. Similarly, in a case-control study, plasma NT-pro-BNP was an independent predictor of the coronary calcium score and carotid intima-media thickness in asymptomatic hypertensive patients [33]. Several prospective studies have recently established a correlation between natriuretic peptides and atherosclerotic disease in the general population. The Heinz Nixdorf Recall and Rotterdam Study reported significant predictive values of NT-pro-BNP for myocardial infarction and stroke [34, 35]. In addition, in the ARIC (Atherosclerosis Risk in Communities) Study, NT-pro-BNP emerged as one of the six markers strongly associated with the incidence of abdominal aortic aneurysms [36]. Notably, this study also identified an independent association between NT-pro-BNP levels and endothelial dysfunction measured by VRI in patients with hypertension.

The strength of this study lies in its ability to address the uncertainties surrounding the relationship between NT-pro-BNP and endothelial dysfunction, an association that has not been investigated in patients with hypertension before. Nonetheless, several limitations should be acknowledged in this study. First, the cross-sectional design prevented the determination of causality between NT-pro-BNP and endothelial dysfunction. Second, the relatively small sample size of hypertensive participants limited subgroup analyses related to comorbidities affecting endothelial function, such as CAD, diabetes, or smoking status. Third, echocardiography was not routinely performed, precluding investigation into the relationship between NT-pro-BNP and early myocardial disease markers. Fourth, potential confounders affecting NT-pro-BNP measurements, such as acute myocardial infarction, sepsis, atrial fibrillation, advanced age, renal failure, and obesity, were not fully accounted for [37, 38, 39, 40]. However, participants with acute coronary syndrome, active infection, and arrhythmia were excluded from the study. Furthermore, serum creatinine levels and body weight did not significantly differ among the three VRI groups. Despite adjusting for age, an important variable associated with VRI, serum NT-pro-BNP levels still independently predicted VRI levels in hypertensive patients. Further longitudinal research is needed to better clarify the causal relationship between NT-pro-BNP and endothelial dysfunction before translating these findings into clinical practice, such as predicting and prognosticating future atherosclerosis in patients with hypertension.

## 5. Conclusions

Natriuretic peptides are pivotal in regulating vascular homeostasis, remodeling, and predicting preclinical and subclinical atherosclerotic disease. This study showed a negative and independent correlation between serum log-NT-pro-BNP levels and VRI values in patients with hypertension. Further prospective studies are needed to elucidate better the causality between serum NT-pro-BNP levels and endothelial dysfunction in patients with hypertension.

## Data Availability

The data presented in this study are available on request from the corresponding author.
